# Natural Polyphenols for Prevention and Treatment of Urinary Tract Infections

**DOI:** 10.3390/ijms24043277

**Published:** 2023-02-07

**Authors:** Maria Maisto, Fortuna Iannuzzo, Ettore Novellino, Elisabetta Schiano, Vincenzo Piccolo, Gian Carlo Tenore

**Affiliations:** 1 Department of Pharmacy, University of Naples Federico II, Via Domenico Montesano 59, 80131 Naples, Italy; 2 Faculty of Medicine, University Cattolica del Sacro Cuore, Largo Francesco Vito, 00168 Rome, Italy

**Keywords:** urinary tract infection, natural polyphenols, uropathogenic *Escherichia coli*, catechins, procyanidins, caffeic acid, quercetin, antimicrobial resistance

## Abstract

Urinary tract infections (UTIs) are the second most common type of bacterial infection worldwide. UTIs are gender-specific diseases, with a higher incidence in women. This type of infection could occur in the upper part of the urogenital tract, leading to pyelonephritis and kidney infections, or in the lower part of the urinary tract, leading to less serious pathologies, mainly cystitis and urethritis. The most common etiological agent is uropathogenic *E. coli* (UPEC), followed by *Pseudomonas aeruginosa* and *Proteus mirabilis.* Conventional therapeutic treatment involves the use of antimicrobial agents, but due to the dramatic increase in antimicrobial resistance (AMR), this strategy has partially lost its therapeutic efficacy. For this reason, the search for natural alternatives for UTI treatment represents a current research topic. Therefore, this review summarized the results of in vitro and animal- or human-based in vivo studies aimed to assess the potential therapeutic anti-UTI effects of natural polyphenol-based nutraceuticals and foods. In particular, the main in vitro studies were reported, describing the principal molecular therapeutic targets and the mechanism of action of the different polyphenols studied. Furthermore, the results of the most relevant clinical trials for the treatment of urinary tract health were described. Future research is needed to confirm and validate the potential of polyphenols in the clinical prophylaxis of UTIs.

## 1. Introduction

**Figure 1:**
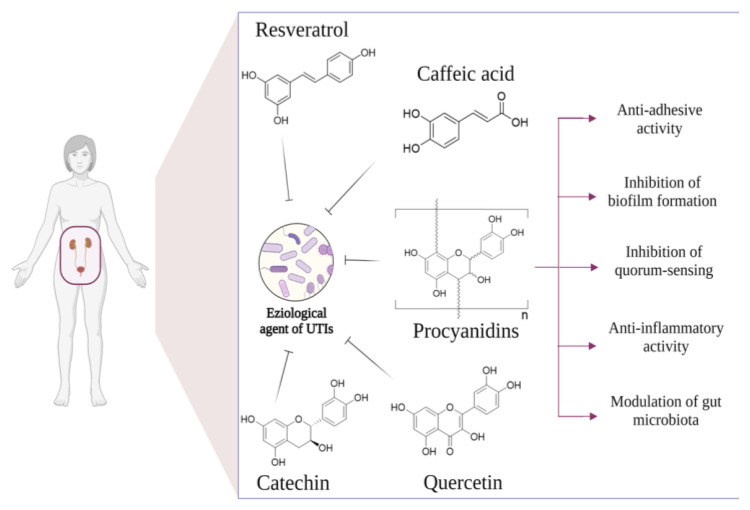
The main studied polyphenols and relative molecular targets for UTI prevention and treatment.

Urinary tract infections (UTIs) are the second most common type of bacterial infection worldwide, with 120–150 million cases diagnosed each year [1]. Based on the anatomical location of the bacterial infections, UTIs can be distinguished differently. They are classified as a pyelonephritis and a kidney infection when the infection affects the upper part of the urinary tract and manifests in severe symptoms such as abdominal pain, fever, chills, flank pain, nausea, and vomiting, which can lead to irreversible kidney damage and sepsis [2]. When infections are confined to the lower urinary tract, urethra, and bladder, UTIs are classified as cystitis and urethritis, characterized by slight symptoms such as dysuria, suprapubic pain, and hematuria [2]. Moreover, UTIs can be classified clinically into uncomplicated UTIs (uUTIs) and complicated UTIs (cUTIs) to distinguish infections of benign origin from those with a higher probability of recurrence or progression to severe pathology [3]. Nowadays, the definition and classification of UTIs is not unique and universally harmonized but is in continuous evolution [1]. Traditionally, uncomplicated UTIs were considered infections in nonpregnant, healthy women that usually resolved with antibiotic treatment, while all other UTIs were classified as complicated, including cystitis in men. Another classification of UTI is recurrent UTI (rUTI). This refers to a new symptomatic infection after appropriate treatment and symptomatic resolution of a previous infection; the most common definition is a frequency of at least two UTIs in 6 months or at least three in a year [4]. It is generally accepted that the major etiological agent UTI is uropathogenic *Escherichia coli* (UPEC), which causes about 85% of cystitis registered in a year worldwide [5]. Specifically, *E. coli* migrates from the gut, where it normally resides, into the urethra and then into the bladder [2]. The migration of *E. coli* from the perianal area into the urethra may be due to poor wiping after a bowel movement, sexual intercourse, or holding urine, as urination helps to flush the bacteria out of the body. In addition to *E. coli*, other Gram-negative bacterial strains, such as *Klebsiella pneumoniae*, *Pseudomonas aeruginosa,* and *Proteus mirabilis*, and some Gram-positive bacteria, including some species of *Staphylococcus* and *Enterococcus*, have been described as additional etiological agents of UTIs [1,6], especially found in immunocompromised and frail patients [7]. Generally, women are more susceptible to bacterial infections than men, mainly due to the lack of prostatic secretion, the short urethra [8], pregnancy, and the easy contamination of the urinary tract with fecal flora. These physiological reasons explain the higher susceptibility of women to UTIs [8]. Despite the fact that UTIs may be a multistrain-dependent infection, the conventional therapeutic approach has not been able to provide specific antimicrobial therapy, but rather mainly relieves symptoms. Recently, due to the dramatic development of antimicrobial resistance (AMR) worldwide [9], there has been a significant limitation in the types of antimicrobial agents that are suitable for efficient UTI treatment [10]. Specifically, some AMR bacteria strains have been isolated in some cases of UTIs, including *Pseudomonas* spp. (carbapenem- and fluoroquinolone-resistant), *Enterococcus* spp. (vancomycin-resistant), and *Enterobacterales* (β-lactamase-resistant) [11,12]. In view of these considerations, there is a growing interest in finding a therapeutic alternative for UTI treatment. Various options such as antiadhesive agents, probiotics, or vaccines have therefore been investigated to prevent these infections [2]. Specifically, in recent years, much attention has been paid to the identification and selection of natural bioactive molecules, especially polyphenols, which are effective in the prevention and treatment of UTIs. Polyphenols are secondary plant metabolites that are widely distributed in fruit, vegetables, and plant-based foods [13]. Based on their chemical structures, natural polyphenols can be divided into five classes, including flavonoids, phenolic acids, lignans, stilbenes, and other polyphenols. A wide range of biological activities is attributed to these molecules, such as antimicrobial, antiproliferative, antioxidant, and anti-inflammatory [14]. In conclusion, the present review summarizes recent findings on the antimicrobial properties of the main studied natural polyphenols (Figure 1) and discusses the mechanisms of actions derived from in vitro and animal- and human-based in vivo studies.

## 2. Proanthocyanidins

Proanthocyanidins (PACs) are oligomers or polymers of monomeric flavan-3-ols with interflavan bonds [13]. Chemically, PACs are classified into two main types, A-type and B-type, which are distinguished by the interflavan linkages [13]. In B-type PACs, the linkage between the successive monomeric units is usually between the C4 position of the upper unit and the C8 or C6 position of the lower unit. In contrast, A-type PACs contain a second ether linkage between the hydroxyl group of the A ring of the lower unit and C-2 of the upper unit [15]. The B-type PAC is mainly found in grapes, green tea, apples, and dark chocolate, while the main natural source of A-type PACs is commonly referred to as cranberries [16]. Due to their high content of A-type PACs, cranberries are the most commonly used natural matrix for the formulation of nutraceuticals and food-based products for the prevention and treatment of UTIs. PACs could interfere with UTIs in humans in several ways. The various mechanisms of action of PACs that have been investigated are inhibition of adhesion of microbial pathogens to uroepithelial cells, reduction of bacterial swarming motility and biofilm formation, reduction of infection-induced inflammation, and finally, potential modulation of the gut microbiota, which in turn could influence the risk of infection in the urinary tract. All of these biological activities are described in detail in the following paragraphs. 

### 2.1. In Vitro Evidence

#### 2.1.1. Antiadhesion Activity

As previously described, UTIs occur when uropathogenic bacteria enter the bladder through the urethra, adhere to the epithelial cell surface, and colonize the tissues [17]. Bacterial adhesion is a crucial step in the spread and development of infection. Thus, the regulation of this process may have a key role in the treatment of UTIs. Specifically, the adhesion of bacteria to urothelial cells is regulated by specific interactions between bacterial surface proteins (adhesins) and receptors on the host cell membrane. The adhesins are located both on microbial surface filamentous organelles, called pili or fimbriae, and on the external membrane of the bacterium [17,18]. The fimbriae-associated adhesins are named lectins, which are also known as carbohydrate-binding proteins, and can interact with a variety of glycoconjugates-proteins on the host cell membrane. Fimbrial adhesins are the main factors associated with the virulence of UPEC and *Pseudomonas Auruginosa*. Generally, UPEC expresses several types of fimbrial adhesins, the most common being type-I-fimbriae (mannose-sensitive) and type-P-fimbriae (mannose-resistant) [19]. Specifically, UPEC with type-I fimbriae are predominantly responsible for bladder infections, while UPEC with P-fimbriae are mostly responsible for kidney infections. Type-I-fimbriae interact with mannose residues of protein receptors expressed on host cells and mediate bacterial adhesion to uroepithelial cells. Structurally, they are filamentous organelles that present a helical rod consisting of repeated subunits of the adhesin FimA that form the main fibrillar structure and terminate in a terminal FimH adhesin, which is directly responsible for bacterial adhesion [20]. Specifically, D-mannose, a simple sugar commonly used in the treatment of UTIs, is able to inhibit UPEC adhesion with fimbriae-1 by binding to the UPEC NH-terminal domain of FimH. On the other hand, P-fimbriae express a galabiose-specific receptor (Gal(α1–4)Gal-) that binds to the glycolipid galabiose, which is expressed on the urothelium of the upper urinary tract, causing mainly pyelonephritis. Structurally, P-fimbriae comprise a helical fiber composite of repeated subunits of PapA adhesins that report a terminal PapG adhesin protein responsible for bacterial adhesion. To investigate the potential antiadhesion activity of PAC, various cell culture models have been developed [21]. In this regard, Nicolosi et el. 2014 studied the antiadhesion activity of procyanidin A2 on pathogenic strains such as UPEC and *Proteus mirabilis* using a human bladder cancer cell line (HT1376) [22]. A significant reduction in the adhesion rate of both UPEC (up to 75%) and *Proteus mirabilis* strains (up to 75%) was observed compared to the control. Other authors, in order to evaluate the direct antiadhesive effect of two potential procyanidin-based products, a cranberry powder (containing 9 mg PACs/g extract), and a PAC cranberry extract on P-fimbriated UPEC designed two experimental in vitro models, the first being a model with primarily cultured bladder epithelial cells, and the second being a model with vaginal epithelial cells. The antiadhesion effect was evaluated before and after exposure to these products. Cranberry powder decreased the mean adherence of UPEC to vaginal epithelial cells from 18.6 to 1.8 bacteria per cell (*p* < 0.001). The mean adherence of *E. coli* to primary cultured bladder epithelial cells was reduced from 6.9 to 1.6 bacteria per cell (*p* < 0.001) in a dose-dependent manner by treatment with 50 ug/mL of PAC extract. Another study conducted by de Llano et at. (2015) evaluated the antiadhesive activity of pure standard cranberry PACs compounds and their microbial metabolites produced at the intestinal microbiota level (such as simple phenols and benzoic acid, phenylacetic acid, and phenyl propionic acids) against UPEC in a bladder epithelial cell culture model. Firstly, the antimicrobial activity and cytotoxicity were tested by evaluating MIC and MTT, respectively. The microbial metabolites tested showed dose-dependent antiadhesive activity against UPEC by reducing the number of bacteria adhering to bladder epithelial cells at all concentrations from 100 to 500 uM, while procyanidin A2 showed a statistically significant reduction (*p* < 0.005) only at 500 uM. Therefore, the authors suggest that the in vitro antiadhesive effect of cranberry PACs against UPEC could be attributed to the microbial metabolites of PACs released at the colonic level, suggesting that their presence in urine could reduce bacterial colonization and progression of urinary tract infections [23]. These studies provide further evidence for the biological relevance of PAC-based cranberry products in the prevention of urinary tract infections. However, further future studies are needed to further elucidate the molecular mechanisms by which cranberry PACs inhibit the adhesion of all bacterial strains involved in the pathogenesis of UTIs.

#### 2.1.2. Inhibitory Activity on Biofilm Formation and Quorum Sensing (QS)

Biofilms are communities of microorganisms attached to the host cell surface by extracellular fibrils and are dispersed in an exopolysaccharide matrix that surrounds them and ensures their cohesion. Specifically, bacteria are irreversibly attached to uroepithelium cells, interface, or each other and are embedded in a matrix of extracellular polymeric substances (EPS) that they have produced. Biofilms are ubiquitous and could be found on a wide variety of sites. They could be produced by a single or multiple bacterial species. Biofilm production was carried out in five consecutive steps which are: initial reversible attachment of planktonic (free state) bacteria to surfaces of uroepithelium; irreversible bacteria attachment to uroepithelium; formation of a complex layer of biomolecules and EPS secretion; maturation of biofilm by acquiring of three-dimensional structure; and, finally, the detachment. Detachment allows bacteria to again take on a planktonic state and could thereby form biofilm in other settings. The biofilm is considered a relevant virulence factor and is currently estimated to be responsible for over 65% of nosocomial infections and 80% of all microbial infections. Due to its peculiar structure, the biofilm is an important physical barrier able to protect bacteria from the action of antibiotics and immune cells [24]. Both Gram-positive and Gram-negative bacteria are capable of forming biofilms. UPEC tends to form biofilm through both type-I-fimbriae and F9-fimbriae [1,25] that mediate the first two steps of the biofilm formation process. Biofilm microbial communities interact with each other through a cell-to-cell communication mechanism, namely, QUORUM SENSING (QS). Particularly, through QS, the microorganisms monitor the bacterial population density resident in the biofilm matrix and the environmental conditions, exchange important information useful for the survival of the entire microbial community, and regulate their swarming activity. This communication occurs through the production and release of signaling molecules called autoinducers. Among these signaling molecules, acyl-homoserine lactones (acyl-HSL) are the most important. Specifically, when bacterial population density is low, the concentration of acyl-HSL in the environment decreases rapidly. Conversely, when population density increases, its concentration increases, and when it exceeds a threshold, acyl-HSL activates a specific bacterial intracellular protein receptor that in turn mediates the expression of some virulence genes. Thus, the potential interruption of the QS communication system and the biofilm formation may be considered potential targets for anti-UTI therapy. To this purpose, various in vitro models are designed to evaluate the potential PAC action on these two virulence factors. Specifically, a recent study [26] described the capacity of methyl gallate-PACs with epicatechin moiety as the terminal unit to reduce the synthesis of acyl-HSL, resulting in an inhibition of microbial communication in the biofilm matrix. Additionally, other authors have described how the treatment with PAC-cranberries extract may inhibit the formation of *Pseudomonas aeruginosa* biofilm. Initially, to determine whether PACs could contribute to enhancing the antibiotic activity of gentamicin, MIC was first determined using different concentrations of PAC compared to different concentrations of gentamicin. PACs showed no direct antimicrobial effect against *Pseudomonas aeruginosa* at any of the concentrations tested, but in combination with gentamicin, they significantly reduce the MIC value of gentamicin from 1.5 µg/mL to 1.3 µg/mL. Moreover, the authors have used crystal violet biofilm staining, resazurin metabolism assays, and confocal imaging to examine the capacity of A-type PACs to disrupt the biofilm produced by *Pseudomonas aeruginosa*. They reported that after the PAC treatment, a valuable reduction of biofilm height and density was observed, with a relevant iron-chelation activity registered [16]. Iron is a key element for the complete maturation of *Pseudomonas aeruginosa* biofilm [27]; thus, its chelation may be a potential therapeutic target. Some studies underline that PACs may exert an iron-chelation activity due to the capacity to coordinate the iron with their several hydroxyl groups. This PAC activity may potentiate the efficacy of traditional antibiotic treatment. In this sense, in an immature biofilm, the bacteria are more prone to antibiotics and immune cells than bacteria in a completed matured biofilm [28]; thus, PACs may be proposed as adjuvant antibiotic molecules. Another study conducted by LaPlante et al. has additionally confirmed the inhibitory effects of PAC-cranberry extract on the biofilm production in bacteria belonging to the species Staphylococcus, with minimum inhibitory concentrations (MIC) ranging from 0.02 to 5 mg/mL and minimum bactericidal concentrations (MBC) ranging from 0.04 to 5 mg/mL [29]. Other authors have investigated the activity of cranberry procyanidins in the prevention of UTIs caused by *Enterococcus faecalis* (*E. faecalis*). In particular, they evaluated the effect of a concentrated commercial cranberry extract formulated in capsules (containing 130 mg of cranberry extract) on the growth and biofilm formation of uropathogenic strains of *E. faecalis* isolated from UTI patient urine. Firstly, the minimum inhibitory concentration (MIC) of the cranberry extract against isolates *E. faecalis* isolates was estimated to be 4.0 mg/mL. Furthermore, it was demonstrated that the tested extract was able to significantly reduce both bacterial survival after 2, 4, 6, and 24 h of treatment (*p* < 0.05) and mass production of biofilms in 100% of *E. faecalis* strains used, resulting in a reduction of the characteristic aggregates of mature biofilms [30].

Another key process for complete biofilm formation is the correct bacterial motility that allows pathogens to correctly adhere to the uroepithelium and colonize surfaces [31]. To this purpose, PACs were also studied as potential inhibitors of bacterial motility. In this regard, O’May and Tufenkji [32] reported that PAC-cranberry extract could block the swarming motility of *Pseudomonas aeruginosa.* These authors designed an in vitro model suitable to study microbial swarming movement. The bacterial motility was studied on a solid surface such as agar plates, simulating the biofilm matrix, where the microbial migration is represented by the tendril formation (a narrow channel in the agar medium). The study showed that under control conditions, *Pseudomonas aeruginosa* forms the tendrils that allow the bacteria to migrate outwards on the agar plate from its inoculum point. However, in the presence of PACs in the medium, *Pseudomonas aeruginosa*, despite the fact that bacteria maintain growth and reproduction rates, does not show tendril formation or other characteristics indicative of swarming activity. The authors hypothesized that the inhibition of bacterial migration was mediated by an interruption of QS communication operated by PAC molecules [32].

#### 2.1.3. PACs Anti-Inflammatory Activity

Another UTI therapeutic target is the treatment of an inflammation state induced by bacterial infections. Specifically, in the infection site, a large number of immune cells, mainly neutrophils, and inflammatory monocytes are recruited, that in turn activate the inflammatory pathways, which finally lead to the massive production of inflammatory modulators such as IL5, IL6, G-CSF, and IL8 [33]. Neutrophils are principally protective and are released into the urinary tract to contrast bacteria involved in the infection process. Only when this response is exaggerated or incomplete could neutrophils determine tissue injury [34]. It is well established that inflammation-related infection is the main cause of UTI recurrence and symptoms [33]. Specifically, patients exhibit symptoms of inflammation including pain, urgency, and increased frequency [35]. Inflammation is also commonly identified in prostate biopsy samples collected from patients with benign prostatic hyperplasia (BPH) [35]. Although pain is not consistently correlated with the presence of BPH, a subset of these patients complains of pain associated with the lower urinary tract.

In this context, the anti-inflammatory role of PACs in the prevention of inflammatory diseases has been widely documented in the scientific literature [36,37]. However, the mechanisms of action related to their potential anti-inflammatory activity in infection sites were not yet fully elucidated. To this end, an in vitro experiment conducted by Huang et al. [38] evaluated the hypothetical mechanism of action of PACs in the modulation of inflammation induced by *E. coli* infections. Firstly, the authors evaluated the MIC of the cranberry methanol extract (CR-ME) on the two different selected *E. coli* strains (ATCC 700336 and ATCC 25922), which was above 256 μg/mL. Since the acceptable concentration for antimicrobial activity of a botanical extract is usually below 50 μg/mL, the tested extract showed no direct antibacterial effect against the two *E. coli* strains. On the other hand, this extract showed a direct effect on inflammation cellular mediators by significantly inhibiting COX-2 activity, with an IC_50_ of 12.8 µg/mL. First, the authors evaluated the MIC of the extract cranberry methanol extract (CR-ME) against the two *E. coli* strains. Based on such promising results, the authors also investigated the CR-ME extract effects on the expression of proinflammatory mediators such as NF-κB in the T-lymphocyte cell line and studied the releasing of cytokines (IL-1β, IL-6, IL-8) on human peripheral blood mononuclear cells. They found that NF-κB transcriptional activation was significantly (*p* < 0.01) inhibited at IC_50_ 12.8 μg/mL, while the cytokine production was significantly decreased at 10 μg/mL CR-ME extract. These data suggest that PACs could reduce the expression of modulators involved in the signal transduction pathway that triggers the inflammatory response following UPEC-induced infection and consequently improve UTI-associated symptoms. However, to better clarify the action of PACs in the modulation of the inflammatory response, the molecular mechanism underlying their activities needs to be further investigated.

#### 2.1.4. Relationship between Gut and Urinary Tract Microbiota

Another recently proposed PAC mechanism of action in the treatment of urinary tract health is related to its ability to modulate gut microbiota. Recently, several authors described a potential link between the health state of gut microbiota and urinary tract wellness. It is now generally accepted that the microbial composition of the urinary tract is completely different from that of the gut microbiome [39]. The altered composition of urinary tract microbiota has been statistically associated with some urogenital disorders [40], while the relationship between UTI and intestinal health state is not completely understood. Some authors hypothesize that this relationship is mainly related to the development of UTIs. As previously reported, UTIs are extraintestinal infections, in which *E. coli*, an intestinal resident and commensal bacteria, invade intestinal cells and migrate into the urogenital tract. In this sense, the intestine could be considered a possible reservoir for *E. coli*, so reducing their invasiveness in intestinal epithelial cells could be a potential means of reducing the risk of UTI [41]. Specifically, Feliciano et al. [41] investigated the influence of cranberry A-type procyanidin-based extract and apple B-type procyanidin-based extract on pathogenic invasion of *E. coli* in enterocytes using an in vitro model of the Caco-2 cell line. The results showed that both the A-type and B-type PACs significantly reduced the migration of *E. coli* into Caco-2 cells in a dose-dependent manner, potentially reducing the invasion of urogenital tissue. A similar in vitro study was conducted by Polewski et al. [42], with the aim to evaluate the potential synergy between A-type PAC-cranberry-based extract and probiotics (*Lactobacillus* species) in reducing the invasiveness of *E. coli* in enterocytes. The results again showed that PACs could significantly reduce the ability of *E. coli* to invade intestinal cells. This result was not observed with probiotics treatment. This led the authors to hypothesize that PACs could interact in a specific way with the fimbriae-associated adhesins expressed on the *E. coli* surface and inhibit their adhesion and subsequent invasion. The above studies seem to indicate an alternative and nondirect approach to UTI prevention, reducing *E. coli* gut load. However, these are very preliminary studies, and further future studies will be needed to support this thesis. 

### 2.2. In Vivo Evidence

On the base of the above-described in vitro antimicrobial procyanidin activity, several clinical trials were conducted to confirm the procyanidin effectiveness for UTI treatment in vivo models. Specifically, one of the most studied procyanidin-based approaches for UTI treatment was the use of cranberry-based products (juice, powder, or nutraceuticals) due to their high title of PACs. The patients that potentially could benefit from cranberry prophylaxis include premenopausal women with frequent but mild UTIs, postmenopausal women with more severe recurrent UTIs, and children and adults with near-permanent and complicated genitourinary malformations with UTIs [43]. One of the first clinical trials that evaluated the efficacy of cranberry juice in the treatment of UTIs was performed by Avorn et. al [44]. This study found that consumption of a low-calorie cranberry juice cocktail (27% cranberry juice, sweetened with saccharin) reduced the incidence of bacteriuria (10^5^ CFUs/mL urine) with pyuria in elderly women with a mean age of 78.5 years. Specifically, this last study has high scientific significance, considering that studies conducted in postmenopausal or elderly women are relatively few. In fact, the majority of available clinical trials reporting the effects of cranberry products for the management of urinary tract wellness were conducted especially in premenopausal or sexually active women. Specifically, in a randomized placebo-controlled study conducted by Tero et Kontiokari et al. [45], 150 premenopausal women with rUTI caused by *E. coli* were recruited to evaluate whether UTI recurrences could be prevented by drinking 50 mL of concentrated cranberry juice. At the end of the 12 months of treatment, a reduction of 20% of recurrence risk in the cranberry group compared to the placebo group was observed. The efficacy of cranberry juice (containing more than 40 mg of PACs per 125 mL) was explored also by Takahashi et al. [46], who conducted a randomized, double-blinded study and demonstrated that the cranberry beverage is superior to placebo in terms of UTI prevention in women over 50 years of age. In this study, cranberry juice prevented the recurrence of UTI in a limited female population after a 24-week intake of the beverage. The possibility to manifest UTI increases significantly in catheterized postsurgery patients. In this regard, Foxman et al. [47] conducted a randomized, double-blind, placebo-controlled study of 160 patients catheterized after elective gynecological surgery involving urinary catheterization. Patients received two capsules of cranberry juice or placebo, two times a day, for 6 weeks after surgery. The results showed that in the cranberry treatment group, the incidence of UTI was significantly lower than in the placebo group. Furthermore, the authors suggest that relevant limitation for the effective use of cranberry juice as standard prophylaxis for UTI treatment is mainly limited to its low and not-standardized PAC content and its low compliance. To overcome this limitation, Vostavola et al. conducted a clinical trial using a nutraceutical formulation based on cranberry polyphenolic extract particularly rich in PACs (56% *w*/*w*). The 182 enrolled patients were randomly assigned to a cranberry powder group (n = 89) or placebo group (n = 93) [48]. The cranberry powder group received 500 mg of cranberry for 6 months and experienced a longer time for the first UTI than the placebo group. In addition, intent-to-treat analyses showed that UTIs were significantly lower in the cranberry group. Despite the encouraging results previously discussed, a recent meta-analyzes study conducted by Fu et al. [49] regarding the efficacy of cranberry in the treatment and prevention of UTIs produced conflicting results due to the extreme heterogeneity of the enrolled population and of the small and unrepresentative number of subjects involved in the studies previously conducted. Furthermore, an additional controversy concerns the clinical efficacy and costs of cranberry supplements, which is probably attributable to the different cranberry products and manufacturer doses [50]. Despite these previous considerations, the efficacy of cranberry-based products was also tested in the pediatric population, producing highly encouraging results. Specifically, these studies have investigated the potential effect of cranberry products, especially fruit juices, on pediatric patients. In a review of eight studies [51], the use of cranberry juice for the prevention of urinary tract infections in healthy children and children with urogenital abnormalities was examined. The results revealed that cranberry products may be an effective option for preventing UTI recurrence in pediatric patients, especially for healthy patients with no anatomical abnormalities. In addition, in another study conducted by Salo and colleagues [52], a total of 263 children aged 1–16 years with normal urinary anatomy or grade I or II vesicoureteric reflux (VUR) were recruited to receive cranberry juice (containing 41 g of cranberry concentrate in 1 L of juice) at a dosage of 5 mL/kg body weight, up to 300 mL per day for a period of 6 months. The intervention did not significantly reduce the number of children who experienced UTI relapses, but it was effective in reducing the actual number of relapses and the use of antimicrobials. The authors underlined that, despite the valuable clinical results obtained, the problem of low compliance remains. Specifically, other clinical trials conducted using cranberry-based products with high doses of PAC have not registered a good acceptance by patients, mainly due to the gastrointestinal disorders that have occurred, with quite high abstinence rates registered (up to 55%), suggesting that cranberry products may not be acceptable for long periods. As a consequence, patients are generally more inclined to use conventional antimicrobial drugs for UTIs treatment, although the long-term treatment could have a negative effect on AMR diffusion [53]. To this purpose, Beerepoot et al. [54] conducted a comparative study to establish the different efficacy of traditional antimicrobial treatment vs. cranberry treatment (capsules formulated with cranberry extract containing 9.1 mg/g of A-type PACs) in 221 premenopausal women affected by UTI. The enrolled women were divided into two groups; the first underwent trimethoprim-sulfamethoxazole (TMP-SMX) treatment, 480 mg per day, and the second group underwent cranberry capsules, 500 mg twice a day. After 12 months of treatment, the TMP-SMX group was found to be more effective than the cranberry group in preventing rUTIs. This study also showed that the mean number and portion of patients with at least 1 symptomatic UTI was higher in the cranberry than in the TMP-SMX group. In addition, the median time to the first symptomatic UTI was 4 months for the cranberry group and 8 months for the TMP-SMX group. Conversely, an increase in resistance to drugs such as trimethoprim, amoxicillin, and ciprofloxacin was observed in isolated *E. coli* cultures after 1 month in the TMP-SMX group, which was not observed in the cranberry group. Similarly, the research team led by McMurdo also highlighted the lower efficacy of cranberry treatment than conventional antimicrobial therapy in their study [55]. In conclusion, the use of cranberry-based products for UTI treatment is particularly controversial. The treatment dosage needs to be assessed and standardized. Further clinical trials should be conducted on a homogeneous enrolled population in order to confirm the effectiveness of a UTI treatment. Considering the increasing AMR diffusion worldwide, and its negative impact on long-term treatment costs [53], cranberry-based products have the relevant advantage of not promoting AMR and, in addition, represent safe remedies even in the high therapeutic doses used [47,56].

## 3. Green Tea Catechins

Green tea catechins obtained from the plant *Camellia sinensis*, commonly known as green tea, are known to possess antioxidative, anti-inflammatory, antitumor, and antiaging properties. The main catechins in green tea are (-)-epicatechin (EC), (-)-epicatechin-3-gallate (ECG), (-)-epigallocatechin (EGC), and (-)-epigallocatechin-3-gallate (EGCG), which have been shown to constitute approximately 30–40% of the water-soluble fraction in green tea [57]. This class of molecules is extensively studied for its antimicrobial activity against a wide variety of microorganisms [58,59].

### 3.1. In Vitro Evidence

Several studies reported the catechin ability to contrast the urogenital bacteria infection evaluated in specific in vitro models. As previously reviewed by Noormandi and colleagues, catechins demonstrated to inhibit the activity of *E. coli*, highlighting the beneficial potential of these compounds for the treatment of UTIs [60]. They underlined how catechins could act as antimicrobial agents against *E. coli* in different ways. These mechanisms of action include the increased production of cytokines (e.g., IL-12 and IL-10), the reduced expression of tumor necrosis factor-alpha (TNF-α) gene, the damage of bacterial cell membrane [61], the bactericidal and antitoxin effects, and the improvement of UTI inflammatory symptoms [62]. As regards the efficacy of polyphenols from green tea for the prevention and treatment of UTIs, it has been underlined that EGC and EGCG are subjected to massive bile extraction, but only EGC showed to be subjected to renal excretion at high rates [63]. In particular, the antimicrobial susceptibility of 80 different *E. coli* strains isolated from UTI cultures were evaluated after the treatment with a green tea aqueous polyphenolic extract tested at different concentrations (0, 2.5, 3.0, 3.5, and 4.0 mg/mL). The obtained results showed that all the strains tested, except one, had minimum inhibitory concentrations (MICs) of ≤ 4.0 mg/mL of green tea extract, with 40% of the isolated strains having an MIC of ≤ 2.5 mg/mL [64]. Since the EGC is the more abundant catechin component in green tea aqueous extract and is also excreted in the urine in a high amount, the authors addressed the role of this compound to potentially be effective as an antimicrobial agent. Conversely, another study proved that EGCG, among the entire green tea catechin collection, represented the most potent antibiofilm agent [65]. The mentioned study showed the ability of EGCG to directly inhibit the biofilm system produced by commensal and uropathogenic *E. coli* by interfering in the development of curli subunits into amyloid fibers, which are important biofilm structural components involved in pathogenetic activities [66]. Therefore, the role of EGCG as a potential adjuvant for antibiotic therapy of biofilm-related infections was suggested. Although the role of a catechin complex or of a single type of catechin in the management of rUTI has been widely described, other authors evaluated the synergic efficacy of catechins in association with other classes of polyphenols or traditional antimicrobial agents on rUTI care. In this regard, a recent study aimed to investigate the synergic combination of protocatechuic acid, catechin, and vanillic acid in the prevention of UTI associated with catheterization [67]. Since the ability of UPEC to adhere to medical surfaces and form biofilms is the main cause of catheter-associated UTI (CAT-UTI), they specifically evaluated how this compound could inhibit this process on silicone surfaces. While protocatechuic and vanillic acid were demonstrated to limit the development of biofilm through the inhibition of bacteria motility and cellular adhesion, catechin showed to affect the cell membrane permeability, potentiating the effects of the other two compounds [67]. In light of these results, a specific combination of dose treatment (0.00162 mol/L of protocatechuic acid, 0.00074 mol l-1 of vanillic acid, and 0.00005 mol l-1 of catechin) was proposed in order to obtain a more effective dose compared to individual compounds. From a similar point of view, studies from the scientific literature have evaluated the ability of catechin to not only directly affect *E. coli* vitality but also to facilitate the activity of other pharmacological remedies, such as traditional antibiotics. As an example, in a study conducted by Passat et al., a total of seventeen *E. coli* specimens were collected from urine specimens of patients with UTI [68]. To evaluate the antibiotic sensibility against twenty-five agents, they used a Kirby Bauer method and calculated the MICs for each antibiotic tested with or without the addition of plant catechin-rich extracts concentrations on two subclasses of the isolated colony, named ED1 and ED2. They found that green tea extract had a synergistic effect with chloramphenicol, amoxicillin, azithromycin, and ciprofloxacin for ED1, and cefodizim for ED2. On the contrary, green tea extract showed an antagonistic effect with amikacin and streptomycin for ED1, and amikacin, gentamicin, tobramycin, streptomycin, cefepime, azithromycin, piperacillin, and kanamycin for ED2. Moreover, the study showed that soluble green tea extract had a synergistic activity with ciprofloxacin among 93.7% of isolated cultures. These results were then justified by the authors based on the assumption that catechin could modify antibiotic resistance by altering the function of essential processes related to the bacterial cytoplasmic membrane, making these microorganisms more susceptible to antimicrobial agents. Particularly, catechin intercalated into microbial phospholipid bilayers and made the microorganisms more susceptible to the antibacterial factors [68,69]. The beneficial potential of catechins on the management of UTI was also explored as regards the synergic combination with other polyphenolic compounds. In this regard, since green tea catechins were demonstrated to be less effective on Gram-negative bacteria [70], a recent study aimed to evaluate the efficacy of nanoemulsions (NEs) of green tea catechins plus cranberry (NE I) and green tea catechins plus curcumin (NE II) to exert antibacterial activity through a microtiter dish biofilm formation assay. As result, both the nanoformulated samples showed to inhibit the growth of uropathogenic-resistant strains, suggesting their use as alternative remedies for the treatment of UTI [70].

### 3.2. In Vivo Studies

Several studies reported on the ability of catechins from green tea in the management of urinary tract disorders, both on animal-based and human-based models. In this regard, Yoon and colleagues tested an innovative nanoformulation based on green tea nanocathechin on an animal model consisting of rats, wherein Chronic Bacterial Prostatitis (CBP) was induced [62]. Nanocatechin was catechin coated with hydroxypropyl methylcellulose by nanotechnology in order to overcome its low intestinal bioaccessibility and bioavailability. Although all the tested treatments (catechin, nanocatechin, and ciprofloxacin) exhibited antimicrobial and anti-inflammatory activity, rats belonging to the nanocatechin group showed a statistically significant decrease in bacterial growth, improvement in prostatic inflammation, and higher catechin plasmatic concentration compared with the catechin group. Therefore, the obtained results may be justified by the higher absorption of the nanoformulated catechin in the body. In addition to this evidence, a study conducted by Lee et al. evaluated the synergic effect of catechin and ciprofloxacin on CBP rats [71]. Specifically, 41 rats were randomly divided into four groups, i.e., the control, catechin, ciprofloxacin, and catechin with ciprofloxacin groups. After a treatment period of 2 weeks, the combination of catechin with ciprofloxacin showed a statistically significant decrease in bacterial growth and improvements in prostatic inflammation compared with the catechin or ciprofloxacin group (*p* < 0.05). Overall, these findings strongly support the use of a synergic combination made of antibiotics and natural-derived molecules as effective supplements in the treatment of UTIs with higher success rates. While the scientific literature is rich in clinical trials conducted on various populations (healthy women, postmenopausal women, subjects after gynecological surgery, older adults, or pediatric patients) treated with different products based on PAC-cranberry, it is relatively poor in data regarding the treatment with other polyphenolic products. A single work, conducted by Kheirabadi and colleagues, aimed to evaluate the treatment with green tea as a supportive treatment to the standard antimicrobial therapy in women with acute uncomplicated cystitis [72]. They found that women treated with green tea (four 500 mg capsules/die) along with the antibiotic medication showed a statistically significant decrease in the prevalence of cystitis-related symptoms together with a significant improvement in the urine test results after 3 days of treatment. These results sparked interest since it was reported that many women with UTIs would prefer using medicinal plants instead of medical help [73]. Nevertheless, further studies are required to confirm this finding.

## 4. Resveratrol

Resveratrol (RSV;3,4′,5-trihydroxystilbene) is one of the most studied stilbenes, mainly found in grape skins, red wine, peanuts, and several woody plants. RSV exists in two stereoisomeric forms cis- (c-RSV) and trans-resveratrol (t-RSV) [74]. RSV has shown many beneficial effects in humans, such as antimicrobial, antiviral, antioxidant, anti-inflammatory, antiaging, anticarcinogenic, and neuroprotective properties [75]. In addition, some studies reported that RSV has a potent inhibitory action on the growth of some human pathogens [76,77].

### 4.1. In Vitro Evidence

In 2006, Wang et al. studied the RSV effect on swarming motility and virulence factor expression of *Proteus mirabilis*, using agar plates with and without the RSV addition [78]. They found that RSV inhibited *Proteus mirabilis* swarming motility and virulence factor expression in a dose-dependent manner. Specifically, RSV significantly inhibited the swarming motility at a concentration of 15 µg ml^−1^ and completely stopped swarming motility at a concentration of 60 µg ml^−1^. The authors explained that the inhibition of swarming motility and virulence factor expression was mediated by the RSV-mediated suppression of rsbA, a His-containing protein, that plays a pivotal role in the regulation of swarming motility and QS. In the same experiment, RSV also inhibited the ability of *Proteus mirabilis* to invade human urothelial cells [78]. Furthermore, Lee et al. evaluated the UPEC antibiofilm activities of t-RSV and some RSV oligomers such as oxyresveratrol, t-stilbene, stilbestrol, e-viniferin, suffruticosol A, and vitisin A [79]. The antibiofilm activities were studied using a crystal violet assay in 96-weel polystyrene plates and confocal laser scanning microscopy (CLSM) and were quantified using COMSTAT analysis. Significant inhibitory effects on biofilm formation were found for t-RSV, oxyresveratrol, ε-viniferin, suffruticosol A, and vitisin A. Among these compounds mentioned, t-RSV and oxyresveratrol showed the highest inhibitory activity on UPEC biofilm formation [80]. Additionally, these two compounds are able to reduce UPEC growth rate by inhibiting FtsZ expression, an essential cell-division protein in prokaryotes [78], that leads to an abnormal and uncontrolled cell elongation of bacterial cells, not followed by a cell division. In addition, the authors underlined that t-RSV and oxyresveratrol reduced the UPEC swarming motility [79] and inhibit the expression of virulence factors, such as the expression of fimbriae-associated genes [79]. These results were observed using scanning electron microscopy (SEM), RNA isolation, and quantitative real-time RT-PCR. Moreover, the same authors also investigated the capacity of t-RSV and oxyresveratrol to inhibit the hemagglutinating ability of UPEC, reducing the UPEC ability to resist the innate immune system. In conclusion, the promising in vitro evidence reported suggests that t-RSV, oxyresveratrol, and RSV oligomers could have potential application in antibiofilm and antivirulence strategies for treating persistent UPEC infections.

### 4.2. In Vivo Evidence

Numerous animal-based studies have evaluated the efficacy of RSV treatment in urinary tract diseases using chronic prostatitis (CP) or UTI in vivo models. CP is a common urologic disorder, with bladder urination dysfunction as the primary clinical manifestation. In a study conducted by Yang and colleagues [81], RSV was used for the treatment of urinary dysfunction in rats with chronic prostatitis (CP). In this study, the bladder pressure and urine volume were monitored. In addition, Western blot analysis was performed to examine the effect of RSV treatment on the expression of some factors implicated in CP pathogenesis, including c-Kit receptor, stem cell factor (SCF), and phosphorylated AKT (p-AKT) in bladder tissue. Results obtained from this study indicated that maximum bladder capacity, residual urine volume, and maximum voiding pressure were reduced in the RSV group compared to the untreated group. The authors showed that RSV is able to improve urinary dysfunction in rats with CP by downregulating the protein expression of SCF, c-Kit, and p-AKT. Similar results were obtained by Zeng et al. [82], who also demonstrated that RSV could improve urinary dysfunction through mast cell suppression and inhibition of TGF-β/Wnt/β-catenin pathway activity. Other studies instead evaluated the RSV effectiveness in the treatment of lower urinary tract symptoms, characterized by bladder-filling symptoms consisting of urinary frequency, nocturia, and flow problems. To this purpose, these studies used animal models with pathological conditions such as type 2 diabetes and obesity, which are important risk factors for UTI [83]. Specifically, Alexandre and colleagues [84] demonstrated that 2-week administration of RVS 100 mg/kg increased the antioxidant activity in the bladder of high-fat diet-fed obese mice. The authors also observed a relevant attenuation of the overactive bladder due to the reduction of contractions during bladder emptying and the reduction of urinary frequency, normalization of detrusor smooth muscle hypercontractility, and restoring urethral relaxation [84]. Furthermore, the high RSV that demonstrated efficacy in the UTI treatment was also related to its ability to reduce the expression of oxidative stress biomarkers. In fact, a study conducted on rats with induced cystitis demonstrated that RSV could improve cystitis-induced bladder dysfunction thanks to its relevant antioxidant potential. Additionally, RSV was shown to reduce bladder epithelial degeneration and lamina propria inflammation in rats at a dose of 10 mg/kg. These effects are due to the ability of this polyphenol to increase glutathione levels and reduce myeloperoxidase activity in the bladder [85]. Other authors have demonstrated that the RSV efficacy in the treatment and prevention of LUTS (lower urinary tract symptoms) may be related to its inhibitory effect on bladder smooth muscle reactivity. They found that RSV at 10 and 100 µM is able to reduce bradykinin-induced rat detrusor contraction in a concentration-dependent manner. This effect was associated with a reduction in cyclooxygenase activity, prostaglandin E2 generation, inhibition of Ca2þ, and activation of L-type Ca2þ channels, which play a crucial role in detrusor contractility [86]. In contrast to a large number of preclinical data, there are not sufficient clinical studies to establish the efficacy of RSV treatment in human UTIs. Furthermore, future studies should be performed in order to assess the RSV effectiveness in human UTI treatment.

## 5. Caffeic Acid

Caffeic acid (CA) is a plant-derived molecule that is classified as hydroxycinnamic acid which contains both phenolic and acrylic functional groups [87]. It is largely contained in various food sources, including coffee, tea, nuts, olive oil, and beer [88]. Its nutraceutical potential is largely demonstrated. CA was studied for its relevant anti-inflammatory, anticancer, antioxidant, and antidiabetic activity. Specifically, CA has been largely employed as an alternative approach to contrast microbial pathogenesis and chronic infection induced by various etiological agents such as bacteria, fungi, and viruses. In the following paragraphs, the application of CA for UTI prevention and treatment, both in in vitro and in vivo systems, was elucidated.

### 5.1. In Vitro Evidence

In 2016, Lima and colleagues studied the CA ability to not only inhibit the growth and vitality of two different bacteria strains responsible to cause UTI (e.g., UPEC 06 (EC06), isolated from urine culture, and *Pseudomonas eruginosa15*(PA15), isolated from the catheter tip), but also its capacity to enhance the activity of conventional antimicrobial drugs [89]. The CA enhancer activity of the traditional antibiotic agents was assayed using the microdilution method of Minimum Inhibitory Concentration. They found that, in *E. coli*, CA showed a synergistic effect when associated with Imipenem, reducing its MIC from 2500 mg/mL to 1574 mg/mL. While, in *Pseudomonas aeruginosa* infection, CA showed a synergistic effect with other two antibiotics, namely, gentamicin, reducing its MIC from 625 mg/mL to 24.61mg/mL, and Imipenem, reducing its MIC from 1250 g/mL to 78.13 mg/mL. Otherwise, CA alone showed weak antimicrobial activity, with an MIC above 1024 mg/mL [90].

### 5.2. In Vivo Evidence

Since the modulation of oxygen-free radical production represents a valuable approach to the treatment of inflammatory diseases, Celik et al. evaluated the capacity of CA-phenethyl ester (CAPE), a compound with proven anti-inflammatory potential in preventing oxidative damage in pyelonephritis (PYN) caused by *E. coli* [90]. The study provided 35 Wistar rats which were grouped into a control group, PYN group, and CAPE group (composite of induced PYN rats treated with caffeic acid). The pathology was induced in the rat by inoculation of *E. coli* (1 × 10^9^ CFU) into the rats in both PYN and CAPE groups via urethral catheterization. The parameters monitored after treatment were malondialdehyde (MDA), nitric oxide (NO), the expression of antioxidant enzymes, catalase (CAT), superoxide dismutase (SOD), glutathione peroxidase (GSH-Px), and xanthine oxidase (XO). After 24, 48, and 72 h of treatment, they found that CAPE administration reduced MDA and NO levels, as well as XO activity, although it increased SOD and GSH-Px activities. Histopathological examination showed that CAPE reduced the inflammation grade induced by *E. coli*. In conclusion, CAPE-based treatment decreases the oxidative damage occurring in PYN and therefore it could be used for the medical management of bacterial nephropathy [90]. CA was also recognized as one of the most representative components of the aqueous extract of Orthosiphon stamineus BENTH. (syn. O. aristatus MIQ.), a plant belonging to the family Lamiaceae and native to tropical Asia. Its use is highly recommended in traditional medicine for UTI symptom alleviation. Sarshar et al. tested its aqueous extract on BALB/c mice presenting transurethral infection with UPEC CFT073. They found that the treatment reduced bacterial growth in the bladder and kidney in a similar manner to those norfloxacin-based therapies [91].

## 6. Quercetin

Quercetin is a polyphenol belonging to the flavonoid class that occurs as a glycoside (with linked sugars) or as an aglycone (without linked sugars). Rich natural quercetin sources are onion, apples, berries, tea, red wine, and kales [92]. This antioxidant molecule was studied in depth in recent years because of its antibacterial and immune-stimulating action, which suggests that it could also help against common bacterial infections affecting gastrointestinal, respiratory, and urinary systems [92]. Specifically, its antimicrobial activity had been demonstrated against numerous bacterial strains involved in UTIs, such as *Staphylococcus aureus*, UPEC, *Salmonella typhimurium*, and *Stenotrophomonas maltophilia*. This wide range of action allows quercetin to be used in treating UTIs alone or in combination with antibiotics [93]. In the following paragraphs, the application of quercetin for UTI treatment, thanks to its microbial suppression in both in vitro and in vivo systems, was illustrated.

### 6.1. In Vitro Evidence

Several authors described quercetin as a potent inhibitor of DNA gyrase, a key enzyme involved in bacterial DNA replication. More precisely, this enzyme is not produced in humans, which makes it an excellent target for antimicrobial agents without causing undesirable side effects in patients. Ohemeng and colleagues tested the quercetin extracted from different natural sources on different bacteria strains implicated in UTIs. They found that quercetin is able to decrease bacteria growth by a hypothesized inhibition of bacterial DNA-gyrase. More recently, this specific mechanism of action was additionally elucidated by other authors who studied the quercetin binding to the gyrase B subunit of *E. coli* DNA gyrase by fluorometric assay [94]. Therefore, considering the above-mentioned severe AMR diffusion, some authors studied the combination of quercetin with different antibiotics (levofloxacin, ceftriaxone, gentamycin, tobramycin, and amikacin) for the treatment of *Pseudomonas aeruginosa* infection [95]. They found that in combination with quercetin, the MICs of the antibiotic tested were drastically reduced (up to 80%). The authors hypothesize that this result may be related to the antibiofilm quercetin activity. As previously described, since the biofilm is the main protective physical bacterial barrier responsible for arresting the penetration of conventional antibiotics, the observed antibiotic MIC reduction may be considered indirect proof of biofilm inhibition formation. As previously described, *E. coli* type-I fimbriae is a significant virulence factor because it is involved in the bacterial bond process on the uroepithelium. Thanks to its adhesion protein, namely, FirmH, *E. coli* could bond the mannose-containing glycoproteins of the uroepithelium determining its colonization [96]. Inhibition of bacterial attachment is an attractive target for UTI treatments. Thus, in a study conducted by Jaiswal et al., 115 phytochemicals from selected plants were studied to evaluate their potential inhibition of UPEC adhesion. This study identified quercetin-3-glucoside as a potential phytochemical, with relevant antiadhesive properties, because of H-bonds forming with the FimH ligand protein [97]. In addition, further studies evaluated the quercetin effectiveness in inhibiting and decreasing polymicrobial biofilm formation by Staphylococcus aureus, *Pseudomonas aeruginosa*, *E. coli*, and *Candida Albicans* on the catheter surface. The inhibition of biofilm formation was detected by the microtiter broth method. Practically, the inhibitory activity to the formation of polymicrobial catheter biofilms at 24 h and 48 h at two different quercetin doses, 53.55 ± 0.01 mg and 50.38 ± 0.01 mg, in comparison to control (nystatin and chloramphenicol) was studied. The results indicate that quercetin treatment is able to degrade polymicrobial catheter biofilms by 46.48% ± 0.01 and damage the polymicrobial biofilm matrix extracellular polymeric substance (EPS) on the catheter. Thus, quercetin seems to have inhibitory activity against the formation of polymicrobial biofilms in catheters and has the potential to be developed as a candidate for new antibiofilm agent against urinary tract infections [98]. Apart from antibiofilm formation activity, quercetin is able to damage the bacterial cell wall ultrastructure and cell membrane integrity of the bacteria [99]. The increased cell membrane permeability makes the antibiotic combination treatment more effective. Furthermore, it was found that quercetin could also modulate the bioavailability of drugs and thus increase the efficacy of the antibiotics [100,101].

### 6.2. In Vivo Evidence

Many promising in vitro results suggested the use of quercetin in several interesting clinical trials. The activity of quercetin in the treatment of UTIs was evaluated in synergy with other ingredients of natural origin with known antimicrobial properties. Specifically, Katske et al. demonstrated that oral quercetin-based supplements were clinically effective, well tolerated, and provided significant symptomatic improvement in patients with clinically proven interstitial cystitis (IC) [102]. Five men and seventeen women were treated for one month with two caps per day of an oral supplement containing 500 mg of quercetin. Symptoms were clinically recorded before and after therapy as well as the patient’s pain assessment (range 0–10). After 4 weeks of treatment, the quercetin supplement was well tolerated and provided significant symptomatic improvement in patients with IC. In 2016, Torella et al. [103] conducted a comparative study to evaluate the effect of treatment with an innovative micellar formulation, consisting of quercetin, curcumin (CS), and hyaluronic acid (HA), in comparison to the traditional intervention with local estrogenic therapy. In total, 145 postmenopausal women were randomly divided into three groups. The first group was treated only with vaginal estrogens; the second group only with HA, CS, curcumin, and quercetin per os; and the third group was treated with HA, CS, curcumin, and quercetin associated with local estrogens. They found that in all the study groups, a significant reduction of recurrent episodes was observed, but in the group treated with the coadministration of both interventions, the effects on the reduction of recurrent episodes was practically doubled. The authors concluded that, in postmenopausal women, the combination of HA, CS, curcumin, and quercetin per os was effective in preventing rUTI, especially in association with vaginal estrogen therapy. A similar association of components was formulated in tablets, for oral administration, and tested on 98 women of reproductive age to evaluate the effect in the prevention of postcoital rUTI. The UTI symptoms significantly decreased after 6 months of treatment, dysuria episodes 2-fold decreased significantly, the number of voiding was reduced (*p* < 0.0001), and, also, the quality of sexual life was improved.

## 7. Other Polyphenols

While the previous paragraphs reported, in detail, the potential applications of the most studied polyphenols for UTI treatment, in this section, information about the polyphenols weakly studied for potential therapeutic application in UTIs is collected. Specifically, Cerezo and colleagues [104] evaluated the antimicrobial activity of the anthocyanin-rich extract, obtained from different blueberry cultivars (Snowchaser, Star, Stella Blue, and Cristina Blue) on different bacterial strains isolated from patients with UTIs. The results showed effective antimicrobial activities of the tested extracts, with observed MIC values ranging from 0.4 mg/mL (for the Stella Blue extract against UPEC of *P. aeruginosa*) to 9.5 mg/mL (for all extracts against UPEC of *Klebsiella pneumoniae*). This study offers the first attempt to understand the potential beneficial effect of blueberry anthocyanins against urinary tract infections, highlighting their strain-specificity on the antibacterial effect [104]. Considering this evidence about the potential application of anthocyanins for the treatment of UTI, a single pilot clinical trial was conducted. In this study, Noce et al. [105] evaluated the effect of hydrolyzable tannins and anthocyanins for the treatment of rUTI in nephropathic patients. In this study, one capsule a day was administered to 16 patients enrolled. The capsule was an oral food supplement based on chestnut extract containing mainly tannins and anthocyanins. Specifically, in each capsule, the polyphenol content was 6.21 mg (4.57 mg of hydrolyzable tannins, 0.94 mg of anthocyanins, 0.51 mg of proanthocyanins, and 0.18 mg of quercetin derivatives). Urinalysis of the sample collected showed a significant reduction of leukocytes in both genders, whereas urinary bacterial flora after the treatment significantly decreased only in male subjects. Tannins seem to exert antimicrobial activity in relation to gender and are mainly effective in counteracting the recurrence of UTIs.

## 8. Polyphenolic Toxicity

Despite the above-documented beneficial effects of polyphenols in UTI treatment, these molecules could also exert some toxicological effects in in vitro and in vivo models. Specifically, some authors evaluated the toxicological effects of catechins in both experimental animal studies and epidemiological surveys, which demonstrated that catechin may cause serious hepatotoxicity in human models. Recently, they also showed that diets containing high amounts (0.5–1%) of catechins deteriorated dextran sodium sulfate (DSS)-induced intestinal inflammation and carcinogenesis [106]. In addition, mice with induced colitis, treated with feed at 1% of catechin, exhibited symptoms of nephrotoxicity, indicated by a marked relevant increase in serum creatinine level [106]. 

Regarding RSV, Vaz-da-Silva et al. registered three relevant adverse events (blood electrolyte changes, nasopharyngitis, and erythematous rash) in 3/24 patients treated with a unique administration of 400 mg of RSV a day [107]. Regarding the toxicological profile of procyanidins, Lluís et al. carried out a limit test to determine the acute oral toxicity and the lethal dose 50 (LD_50_) and some genotoxicity tests of the polyphenolic compound contained in a marc extract (titled at 74.8% in procyanidins) in rats. They found that LD_50_ observed was higher than 5000 mg/kg, and, at doses up to 2000 mg/kg, it did not show an increase in terms of micronucleated erythrocytes after 72 of treatment [108]. On the other hand, others also demonstrated that nutraceutical formulations based on apple or grape procyanidins demonstrated good safety and no genotoxicity, evaluated according to the Ames test [109]. Among all the polyphenols mentioned in this text, the toxicological profile of quercetin was the most investigated. Specifically, in the in vitro model, quercetin showed remarkable mutagenic activity in most standard strains of *Salmonella typhimurium*, reverse mutations, and DNA single-strand breaks in *E. coli*, irrespective of metabolic activation. In addition, quercetin did not exhibit any mutagenicity forward mutation assay alone or with a bioactivation system in two *Bacillus subtilis* strains [110]. The quercetin mutagenic activity shown in bacteria models was confirmed by experiments conducted on eukaryotic cells, including yeast cells, at relatively high concentrations (up to 10 mg/incubation mixture) [110]. The Ames assay, conducted with quercetin, showed that the methylation of quercetin at the different hydroxyl groups could significantly attenuate or completely suppress its mutagenic activity [110]. Interestingly, all the above-mentioned in vitro quercetin toxic activities were not confirmed by in vivo experiments. Specifically, in the mice and rats model, the quercetin oral administration did not induce any relevant mutagenicity/genotoxicity effects [110].

## 9. Materials and Methods

### Search Strategy

A comprehensive literature search was conducted using the PubMed-NCBI and Google Scholar database listings for relevant publications. The combinations of the following keywords were used: “nutraceuticals,” “polyphenols,” “bioactive compounds,” “urinary tract infection”, “biofilm formation,” “adhesion,” “mechanisms of action,” and “targets of action.” A thorough screening of scientific literature databases was conducted for the section on the evidence from clinical trials. We exclusively included (i) clinical trials from the last 15 years, with a single exception for a study, conducted in 1994, which we cited for its historical relevance; (ii) studies evaluating the effects of treatment with food-derived bioactive polyphenol formulated both in nutraceuticals and food products, excluding articles with diet-intervention studies; and (iii) studies reporting the effect of exclusive nutraceutical supplementation, excluding those investigating the administration with pharmaceutical treatments.

## 10. Conclusions

In this review, the most relevant studies regarding the potential application of polyphenols for UTI prevention and treatment were reported. While the scientific literature is particularly rich in original papers, reviews, and metanalysis studies regarding the effects of cranberry procyanidins in the management of UTI, less documented are the effects of the other polyphenols class in this type of disorder. To this end, the most relevant papers related to in vitro evidence are summarized, underling the possible molecular target implicated in UTI treatment. In addition, exhaustive research about the most relevant clinical trial conducted using nutraceutical or food-based products were reported, and the results are also summarized in Table 1. Further investigations are needed to elucidate the mechanism of action of most polyphenols studied, and an essential study of dose assessment is highly required to standardize the application of natural polyphenols in UTI treatment.

## Figures and Tables

**Table 1:** Most-relevant published clinical trials from the last 15 years reporting effects on UTI treatment after supplementation with food-derived polyphenols.

Trial Type	Subjects	No. of Subjects	Age (Years)	Treatment(s)	Duration of Treatment	Main Outcomes	Ref.
Procyanidins							
R, DB, PC	Elderly Woman	153	78.1 ± 8.3 (Cranberry group) 79.0 ± 9.4 (Placebo group)	300 mL per day of a commercially available standard cranberry beverage (containing 27% cranberry juice) 300 mL per day of a specially prepared synthetic placebo drink	6 months	42% of the pathologic bacteriuria (*p* < 0.01) compared to the control group and 27% odds of remaining bacteriuric-pyuric (*p* < 0.01) compared to the control group	[44]
R, DB, PC	Premenopausal women	150	32 ± 9.8 (Cranberry group) 30.0 ± 11.8 (Lactobacillus group) 29.0 ± 10.5 (Control group)	100 mL of lactobacillus drink (containing 4 × 10^10^ cfu of Lactobacillus GG/100 mL) five days a week for one year (Lactobacillus group) 50 mL of cranberry–lingonberry juice (containing 7.5 g cranberry concentrate and 1.7 lingonberry concentrate in 50 mL of water)	6 months	−20% of recurrence (*p* < 0.05) compared to the control group	[45]
R, DB, PC	Women	300	Over 50	125 mL placebo juice once daily, before sleeping. Group P 125 mL cranberry juice (containing > 40 mg proanthocyanidin per 125 mL) once daily, before sleeping. Group A	24 weeks	29.1% of patients showed UTI relapse in group A 49.2% of patients showed UTI relapse in group P, (log-rank test; *p* = 0.0425)	[46]
R, DB, PC	Subjects undergoing elective gynecologic surgery	160	>18 years old	2 cps of cranberry juice (equivalent to two 8-ounce servings of cranberry juice) twice per day 2 cps of placebo g 2 times per day	6 weeks after surgery	Lower UTI occurrence in the cranberry treatment group compared to the placebo group (15/80 patients (19%) versus 30/80 (38%)	[47]
R, DB, PC	Woman with recurrent UTIs	182	55.3 ± 13.3 year (active group) 55.1 ± 10.9 year (placebo group)	Placebo 500mg of cranberry powder	6 months	Cranberry group, the UTIs were significantly fewer (10.8% vs. 25.8%, *p* = 0.04) Cranberry group experienced a longer time to first UTI than the placebo group (*p* = 0.04)	[48]
R, DB, PC	Children with normal urinary anatomy or grade I or II VUR	263	1–16 years old	Placebo juice 5 mL/kg body weight cranberry juice	6 months	−6 days on antimicrobials per patient-year; 95% CI, −7 to −5; *p* < 0.001)	[52]
R, DB	Premenopausal women with recurrent UTIs	221	18 years or older	480 mg trimethoprim-sulfamethoxazole (TMP-SMX) once daily cranberry caps 500 mg twice daily	12 months	78.2% vs. 71.1% patients with at least 1 symptomatic UTI, cranberry vs. TMP-SMX group	[53]
R, DB	Women with two or more antibiotic-treated UTIs in the previous 12 months	137	≥45 years	100 mg trimethoprim cranberry extract 500 mg	6 months	Time to first recurrence of UTI was not significantly different (log-rank test: Δ = 2.7, χ2 (2.7, 1) *p* = 0.100) 84.5 days median time to UTI recurrence (cranberry group), 91 days for trimethoprim group (U = 166, *p* = 0.479).	[55]
Catechins							
R, SB, PC	Premenopausal nonpregnant women with acute uncomplicated cystitis	70	18–50 years	Placebo powder + 480 mg tablets of co-trimoxazole twice daily for three days (placebo group) Green tea catchins (four 500 mg caps/die) + 480 mg tablets of co-trimoxazole twice daily for three days (green tea group)	3 days	Green tea group exhibited significant improvement in urinalysis data (abnormal urine color, pyuria, and bacteriuria) among with Placebo group, except for hematuria After 4 weeks, 2.86% of patients in the green tea group, and after 6 weeks, 15.38% of patients in the placebo group had the symptoms of recurrent cystitis	[72]
Quercetin							
CT	Subjects with documented interstitial cystitis	22	53.1 years	1 caps of Cysta-Q complex (containing 500 mg of quercetin) twice daily	4 weeks	From 11.3 +/− 0.6 to 5.1 +/− 0.7 (*p* = 0.000001) improved the mean problem index From 11.9 +/− 0.9 to 4.5 +/− 0.5 (*p* = 0.000001) mitigated the mean symptom index From 8.2 +/− 0.4 to 3.5 +/− 0.4 (*p* = 0.000001) the mean global assessment score ameliorated	[102]
MC, CT, PS	Postmenopausal women with recurrent UTIs during the last year	145	Group 1: 56.4 ± 3.2 Group 2: 56.6 ± 2.9 Group 3: 57.0 ± 4.1	Group 1: Vaginal estrogens (0.005% estriol vaginal gel, daily for three weeks and then twice weekly up to 12 weeks; repeat treatment every three month) Group 2: HA, CS, curcumin and quercetin *per os* ((2 capsules daily for 15 days a month for 3 months, then one capsule daily for 15 days a month for the next 9 months) Group 3: HA, CS, curcumin and quercetin associated with vaginal estrogens	12 months	After 6 months, in group 3 was reduced the recurrent UTIs episodes compared to those receiving single treatments (group 1 and group 2)	[103]
Tannins							
CT, PS	Nephropathic patients affected by recurrent UTIs	26	>18 years old	Oral supplementation (containing 4.57 mg of hydrolyzable tannins, 0.94 mg of anthocyanosides, 0.51 mg of proanthocyanidins, 0.18 mg of quercetin derivatives) Untreated subjects (control group)	6 weeks	In supplemented group a significant reduction in urine leukocyte content was observed. Urinary bacterial flora decreased significant untreated weeks of treatment vs. untreated subjects	[105]

Abbreviations: R: randomized, CT: clinical trial, PS: pilot-study, MC: multicentric, PC: placebo-controlled, DB: double-blind, SB: single-blind.

## Data Availability

Not applicable.
